# Development and validation of a questionnaire to measure the congenital heart disease of children’s family stressor

**DOI:** 10.3389/fpubh.2024.1365089

**Published:** 2024-05-01

**Authors:** Yi Zhang, Hang Zhou, Yangjuan Bai, Zhisong Chen, Yanjiao Wang, Qiulan Hu, Mingfang Yang, Wei Wei, Lan Ding, Fang Ma

**Affiliations:** ^1^Department of Nursing, Kunming Municipal Hospital of Traditional Chinese Medicine, Kunming, China; ^2^Department of Clinical Psychology, Yunnan Provincial Hospital of Infectious Disease, Kunming, China; ^3^Cardiology Department, The First Affiliated Hospital of Kunming Medical University, Kunming, China; ^4^Psychiatric Department, The First Affiliated Hospital of Kunming Medical University, Kunming, China; ^5^ICU in Geriatric Department, The First Affiliated Hospital of Kunming Medical University, Kunming, China; ^6^Urology Department, The First Affiliated Hospital of Kunming Medical University, Kunming, China; ^7^Neurosurgery Department, The First Affiliated Hospital of Kunming Medical University, Kunming, China; ^8^General Surgery Department, The First Affiliated Hospital of Kunming Medical University, Kunming, China; ^9^Department of Nursing, The First Affiliated Hospital of Kunming Medical University, Kunming, China

**Keywords:** congenital heart disease, validity, reliability, family, stressor

## Abstract

**Background:**

Families of children with congenital heart disease (CHD) face tremendous stressors in the process of coping with the disease, which threatens the health of families of children with CHD. Studies have shown that nursing interventions focusing on family stress management can improve parents’ ability to cope with illness and promote family health. At present, there is no measuring tool for family stressors of CHD.

**Methods:**

The items of the scale were generated through qualitative interviews and a literature review. Initial items were evaluated by seven experts to determine content validity. Factor analysis and reliability testing were conducted with a convenience sample of 670 family members. The criterion-related validity of the scale was calculated using scores on the Self-Rating Anxiety Scale (SAS).

**Results:**

The CHD Children’s Family Stressor Scale consisted of six dimensions and 41 items. In the exploratory factor analysis, the cumulative explained variance of the six factors was 61.085%. In the confirmatory factor analysis, the six factors in the EFA were well validated, indicating that the model fits well. The correlation coefficient between CHD Children’s Family Stressor Scale and SAS was *r* = 0.504 (*p* < 0.001), which indicated that the criterion-related validity of the scale was good. In the reliability test, Cronbach’s α coefficients of six sub-scales were 0.774–0.940, and the scale-level Cronbach’s α coefficient value was 0.945.

**Conclusion:**

The study indicates that the CHD Children’s Family Stressor Scale is valid and reliable, and it is recommended for use in clinical practice to assess CHD children’s family stressors.

## Background

Congenital heart disease (CHD) is the most common birth defect worldwide, accounting for almost 30% of all major congenital disabilities, and the mean prevalence of CHD globally was 8.2 per thousand (1, 2). CHD has become the leading cause of death from birth defects in infants and young children (3–5), and the global age-standardized mortality rate of CHD in 2017 was 3.9/100,000 (5). Compared with normal children, children with CHD are prone to recurrent disease with many serious complications. Some children with complex CHD often need multiple palliative or corrective surgery, continuous nutritional support, and drug treatment (6–8). Furthermore, children with CHD might have lower cardiorespiratory endurance and physical activity, impaired growth and personality development, intellectual and learning disabilities, and psychiatric disorders such as anxiety, depression, and attention deficit (9), all of which cause a huge burden to the family. It has been evidenced that parents of children with CHD have to deal with the stress of changing parental roles, parenting burdens and balancing responsibilities, communication problems with healthcare providers, insufficient support network, financial issues and balancing work and family responsibilities (10). As for the children with CHD, they might suffer peer bullying and isolation, difficulties with academic achievement, distressing inability to participate in sports and disturbed body image (6). In addition, for the siblings of children with CHD, their education, social activities, and physical and mental health are affected as well (11, 12). All the above suggest that families of children with CHD are under great pressure, which will lead to adverse health outcomes for family members and family function as well, and ultimately reduce the family health level (13–15). Therefore, it is particularly important to take action to relieve the stress of families of children with CHD. Research has shown that nursing measures focusing on family stress management can reduce parents’ distress, improve their ability to cope with the disease (16–18), and promote family health. The first step to conduct family stress management is an accurate diagnosis of family stressors. Therefore, the development of the stressors scale for families of children with CHD can help healthcare providers accurately and efficiently assess the stressors in children’s families, and based on this, interventions and educational strategies can be established to help families effectively cope with stress, thereby promoting family health (19).

A wide variety of measures, including general and disease-specific scales, have been used to assess parental stressors. Streisand et al. (20) developed the Pediatric Inventory for Parents (PIP) to measure parental stressors consisting of 42 items and four dimensions. PIP have been applied in many chronic diseases, including cancer, inflammatory bowel disease and type I diabetes, and demonstrated good reliability and validity (21). Katie et al. (22) developed the Pediatric Parenting Stress Inventory (PPSI) based on a literature review and clinical experience to identify problems and disease-related stressors experienced by parents of children with severe illness, which consists of 45-item and five dimensions. Cronbach’s α of the scale is 0.94 and has good validity. Miles et al. (23) developed the Parental Stressor Scale: Pediatric Intensive Care Unit (PSS:PICU) to measure the stressors of parents when their children were admitted to the intensive care unit, which includes 79 items and seven dimensions. Cronbach’s α of the scale ranges from 0.69 to 0.95 for each dimension and has good validity. Margaret et al. (24) developed the Parental Stressor Scale: Neonatal Intensive Care Unit, which is designed to measure parental perceptions of stressors arising from the physical and psychosocial environment of the neonatal intensive care unit. The scale includes 26 items and three dimensions, and Cronbach’s α of the scale ranges from 0.89 to 0.94. Miles et al. (25) also developed the Parental Stressor Scale: Infant Hospitalization (PSS:IH) based on Margaret’s NICU parental stressor scale to measure the stressors of parents of children in the ICU or general pediatric ward. It includes 22 items and three dimensions, and Cronbach’s α of the scale ranges from 0.87 to 0.90. However, the above scales focused on the parents instead of the family, ignoring the systemic responses of the family as a whole. Burke et al. (26) obtained The Burke Assessment Guide to Stressors and Tasks in Families with a Child with a Chronic Condition by studying nine families for 10 years and is gradually perfected by compiling the family’s description of the problems dealt with in the past and the problems dealt with now. Finally, 52 concerns of families of children with chronic diseases were summarized, including 11 groups of different stressors, which were used to evaluate the stressors of families with chronic diseases. However, it was only used as an evaluation tool and did not form a scale, and its reliability and validity were not yet clear. So far, there is no tool to assess stressors in families of children with CHD. The lack of a well-validated and reasonably comprehensive scale to measure the stressors of families with children diagnosed with CHD may be a key barrier to implement family stress management for these families. The purpose of this study was to develop and validate a scale to assess the stressors of families with children diagnosed with CHD using the family as the research unit.

## Methods

### Study design

The scale was developed using a sequential exploratory mixed research method combining qualitative research with a quantitative procedure in three phases: Phase 1 for generation of the item pool through interviewing family members of children with CHD and reviewing the relevant literature; Phase 2 for item improvement and content validity evaluation by expert judgments; Phase 3 for evaluation of the psychometric properties, such as construct validity, internal consistency reliability and criterion-related validity. The study was approved by the Kunming Medical University Research Ethics Committee. Participants in both qualitative and quantitative research understood the purpose of the research, participated voluntarily in this research, and had the right to withdraw from the research at any time.

### Phase 1: Generation of the item pool

#### Procedure and participants

A qualitative study using in-depth, face-to-face interviews was applied to explore the stressors in families of children with CHD. We used a purposive sampling method to select family members of CHD children in a tertiary referral hospital in Yunnan Province, China. Participants were stratified according to the types of surgery (interventional medical and surgery operation) of CHD children and then sampled into subgroups divided according to the different stages of the disease (diagnosis, preoperative, postoperative, and follow-up). The family members were selected based on (1) having a son or daughter with CHD who was between 0 and 14 years of age, (2) having the ability to understand and express their own experiences and ideas in Chinese, and (3) being able to participate in the study with a signed informed consent form. The exclusion criteria were as follows: family members with complicated conditions such as severe diseases of other systems except for heart or other serious diseases or already being in end-stage CHD. A total of 21 participants (five fathers and 16 mothers) completed semi-structured interviews. Examples of guiding questions were as follows: (a) What are you worried about since the child was diagnosed? (b) What are the differences in your family life before and after your child has got the illness? (c) Apart from the illness of the child, what are other difficulties for your family? (d) What makes you feel stressed in the process of treating the disease and caring for the child? (e) What difficulties do family members face when working and socializing? (f) Please describe the most memorable difficulties you experienced after your child was diagnosed. The data were analyzed using directed content analysis according to the double ABCX model of family stress and adaptation (27, 28). In the model, the stressors of the family are cumulative and include five aspects: the initial stressors and their hardship, normative transitions, the prior strains, the consequences of a family effort to cope, and ambiguity, both intra-family and social, which could inform the initial direction of qualitative data analysis without limiting the identification of new themes (28, 29).

Content analysis was selected as the preferred data analysis method to obtain themes. The themes obtained from qualitative research analysis provided a theoretical basis for the formation of the scale’s structure and were combined with relevant literature to compile the scale’s item pool. To eliminate the risk of bias, the interviewer (ZY) and corresponding author (MF), who had expertise in qualitative data, analyzed the data independently. Finally, 10 themes emerged from qualitative research; Table 1 shows the themes of the qualitative study.

**Table 1:** Themes of qualitative study.

Domain	Theme
The initial stressor and associated hardships	Confusion regarding the disease
	Hardships encountered during treatment of the disease
	The heavy financial burden
	The unusual growth track of the child due to the disease
Normative transitions	Normal events becoming abnormal for the family
Prior strains	Impaired family functioning
The consequences of family efforts to cope	Family vulnerability
	Family resilience
Intrafamily and social ambiguity	Family boundary ambiguity induced by role alteration
	A lack of knowledge about community support
Sociocultural values	Family stigma

DeVellis (30) suggested that the item should avoid double-barreled, ambiguous pronoun references and exceptionally lengthy items to assure its clarity and reading difficulty level. A combination of forward and reverse entries was used to avoid an acquiescence, affirmation or agreement bias (30). Entries retained a degree of redundancy so that items that did not behave as expected could be removed at a later stage of validation (30). A total of 85 items were generated based on the results of qualitative research and literature review.

### Phase 2: Item improvement and content validity evaluation

#### Procedure and participants

Two rounds of item improvement were performed during this phase. The first round applied expert group meetings. We invited two experts who had been engaged in children’s heart treatment and worked for 15 years, one expert in the field of children’s nursing who had worked for 15 years, one expert in the field of child psychology with PhD qualification and worked for 15 years, one expert in the field of CHD health management and worked for 20 years, one social worker working for CHD relief programmer and worked for a year to hold an expert consensus meeting. Each expert indicated their own suggestions (to add, delete, and modify) on whether the items were clearly stated, repeated, reflected the stressors of families with children diagnosed with CHD, and were suitable for the Chinese cultural background. The second round used expert consultation. An e-mail consultation questionnaire was sent separately to two experts in the field of children’s heart treatment and five experts in the field of children’s heart nursing. These experts included one pediatric cardiac surgery specialist with PhD qualification who worked for 10 years; one pediatric cardiology specialist with a bachelor’s degree and who worked for more than 20 years; two CHD nursing specialists with master’s qualifications who worked for 15 years; three nursing specialists with bachelor’s degree and worked for more than 20 years in CHD caring. The experts were asked to rate the items on the scale using a 4-point evaluation scale (1 = not relevant, 2 = somewhat relevant, 3 = relevant, and 4 = highly relevant) (31). There is an opinion column in the consultation questionnaire, and experts can recommend additions, deletions or revisions to dimensions and items. Through calculating the inter-rater agreement (IR); content validity index for items (I-CVI); scale-level content validity index and universal agreement calculation (S-CVI/UA); scale-level content validity index, averaging calculation method (S-CVI/Ave), and the content validity of the scale was evaluated (31, 32). Items with the I-CVI value of ≥0.78 were retained (31).

After the expert consensus meeting discussion, the initial item pool was revised from 85 to 76, eight items were deleted, four items were modified, and one item was added. In the expert consultation, the content validity of the calculation scale is as follows: 63 items with the I-CVI of 1.00, 12 with the I-CVI of 0.71, and 1 with the I-ICV of 0.57. Finally, 13 items with the I-CVI of ≤0.78 were deleted according to the evaluation criteria of I-CVI. Based on the results of the expert consultation, no additions, deletions, or revisions to the scale items were required IR = 0.83, indicating good agreement among experts; The S-CVI/UA was 0.829, and the S-CVI/Ave was 0.949, which meant items had good correlation and representativeness, suggesting the scale can be applied in practice. Responses were given a 5-point Likert scale (0 = no effect, 4 = extremely affected) to reflect the impact of the items that occurred in the family, and the non-occurrence in the scale options with a score of 0 indicates that the item does not occur. A total of 20 family members of children with CHD were selected to conduct a pilot study in the form of interviews to find out whether the difficulty level of the item was appropriate and whether the expression was accurate and clear. They generally responded that the scale was easy to fill in, and the items were clear and unambiguous.

### Phase 3: Psychometrics properties of the questionnaire

#### Questionnaire

The questionnaire is divided into four parts. The first part is the introduction, which explains the research purpose, confidentiality principle, and ethical principle. The second part is the general data questionnaire, which collects relevant demographic data, including age, gender, role of family members, educational level, diagnosis of the child, and type of surgery. The third part is the 63-item CHD Children’s Family Stressor Scale. The fourth part is the Self-Rating Anxiety Scale (SAS). Studies have shown that the more stressors people have, the more serious their anxiety (33) are. Therefore, in this study, the anxiety level of the children’s family members was used to calculate the scale’s calibration-related validity.

#### Sample

We gave out the CHD Children’s Family Stressor Scale to family members of children with CHD based on the following inclusion and exclusion criteria. The inclusion criteria are as follows: (1) children with CHD are less than 14 years old; (2) children’s stem family members, such as parents, grandparents, and siblings (over 18 years old); (3) living with the child; (4) agree to participate in this study; (5) were not diagnosed with psychiatric, with basic listening, speaking, reading, and writing skills, and being able to read Chinese. The exclusion criteria are as follows: (1) history of mental illness or cognitive impairment, such as psychotic, mood, or anxiety disorder; (2) children with complicated conditions, such as severe diseases of systems other than the heart or end-stage CHD; (3) communication barriers (unable to communicate in Chinese). The sample size was calculated based on an item (an item is a question within the questionnaire) to participant ratio of 1:5–1:10, and an average attrition rate of 20% in questionnaire responses was considered (30).

#### Data collection

The survey was conducted in three tertiary hospitals in China from December 2021 to March 2022. These tertiary hospitals are located in cities southwest China, such as Kunming, Chengdu and Guangzhou. The researchers collected data through paper questionnaires and the Questionnaire Star app. The paper questionnaire and the electronic questionnaire were sent by the researcher to the participants to fill in. The researchers will explain the purpose of the study and the rules for filling it out to participants. Questionnaire completion was voluntary and anonymous. Each questionnaire was completed independently by participants. A total of 800 questionnaires were sent to the participants, and 748 questionnaires were returned. The response rate of the questionnaire was 93.5%. After excluding unqualified questionnaires, a total of 670 questionnaires were recovered and analyzed. The criteria for excluding are as follows: (1) missing items are greater than or equal to one item, (2) all items have the same score, and (3) select two or more options for the same item.

#### Data analysis

The database was established by Excel and imported into R language, SPSS 26.0 statistical software, and AMOS 24.0 statistical software for data analysis. First, exploratory factor analysis (EFA) and confirmatory factor analysis (CFA) were used to test the construct validity of the scale. The entire sample was randomly divided into two sub-samples (A and B). Sub-sample A (*n* = 335) was used for EFA, and sub-sample B (*n* = 335) for CFA. Before using EFA, the Keizer–Meyer–Olkin (KMO) measure of sampling adequacy and Bartlett’s sphere test were conducted to check whether the data were suitable for EFA. Data factorability was determined by a KMO value of at least 0.60 and the significance of Bartlett’s test of sphericity value (*p* < 0.05) (34). A Monte Carlo parallel factor analysis was performed using R language to determine the number of factors. SPSS 26.0 was used to perform principal axis factor analysis and oblimin rotation method to identify meaningful factor dimensions (29, 35). The appropriate items were selected based on the factor loading more than 0.4 (36, 37). Sub-sample B (*n* = 355) was used for CFA to verify the feasibility of factor results from EFA with AMOS 24.0. Chi-square/degrees of freedom (CMIN/DF), incremental fit index (IFI), comparative fit index (CFI), goodness-of-fit index (GFI), root of mean square residual (RMR), and root-mean-square error of approximation (RMSEA) was adopted as goodness-of-fit indicators to evaluate the degree of interpretation of the constructed factor structure to the sample (38, 39). The scale’s calibration-related validity was calculated using scores on the Self-Rating Anxiety Scale (SAS). Cronbach’s α coefficient was used to evaluate the internal consistency reliability, and the acceptable level should be greater than 0.7 (40). The configural invariance, metric invariance and scalar invariance models were performed to verify the measurement invariance of the scale with AMOS 24.0.

## Results

### Demographic data

Of the 670 family members who submitted eligible questionnaires, 291 (43.4%) were fathers, 355 (53.0%) were mothers, 10 (1.5%) were grandmothers, three (0.4%) were grandfathers, five (0.7%) were brothers and five (0.7%) were sisters. The age ranged from 18 to 60 years old, with an average age of 33.43 years. A total of 89 (13.3%) had baccalaureate degrees or above, and 581 (86.7%) had associate degrees or below. In total, 90 (13.4%) children had not received surgery, 299 (44.6%) underwent medical intervention operation, and 281 (41.9%) received surgery. The age of children ranged from 0.2 to 14 years old, with an average age of 5.9 years. Overall, 85 (12.7%) children had complex CHD, 338 (50.4%) had simple CHD and 247 (36.9%) were not clear about their specific diagnosis. Table 2 shows the characteristics of the study participants.

**Table 2:** Characteristics of study participants (*N* = 670).

	Characteristics	Frequency	Percentage
Family roles	Mother	355	53.0
	Father	291	43.4
	Grandfather	3	0.4
	Sister	5	0.7
	Brother	5	0.7
	Grandfather	3	0.4
Age of family member, years	18–20	3	0.4
	21–40	543	81.0
	41–60	101	15.1
	>60	23	3.4
Education of family member	Primary school	177	26.4
	Junior high school	257	38.4
	High school	147	21.9
	Bachelor’s degree	89	13.3
Age of children, years	<1	52	7.7
	1–3	185	27.6
	4–7	201	30.0
	8–14	232	34.6
Surgical type of children	Not surgery	90	13.4
	Medical interventions	299	44.6
	Surgical operations	281	41.9
Children’s disease complexity	Complex	338	50.4
	Simple	85	12.7
	Unknown	247	36.9

### Exploratory factor analysis

EFA was performed on 63 items. In the EFA group, all the KMO values were more than 0.6, and the *p*-values for Bartlett’s test of sphericity were < 0.001, suggesting the data were appropriate for factor analysis (34). A six-factor solution including 41 items and explaining 61.085% of the total item variance in the database was obtained. Factor 1 (seven items), “ambiguity, both intra-family and social” accounted for 9.324% of the variance; Factor 2 (eight items), “enduring treatment ordeals” accounted for 12.351%; Factor 3 (10 items), “dilemma in the family coping process” accounted for 13.014%; Factor 4 (four items), “confusion in the understanding of the disease” accounted for 6.583%; Factor 5 (four items), “family resilience” accounted for 9.378%; Factor 6 (eight items), “unusual growth track of the child owing to the disease” accounted for 11.983%. Table 3 shows the loadings of items in each factor.

**Table 3:** Rotated factor loadings of the family stressor scale for children with CHD items.

Item	Factor loadings	Variance explained	Cronbach’s α
Factor 1: Ambiguity, both intra-family and social		9.324%	0.906
52. Sibling’s complaint about being treated differently	0.796		
36. Child being talked about at school because of the illness	0.742		
61. Unable to obtain professional help	0.731		
63. Family being treated differently because of children’s illness	0.685		
59. Not act as a qualified parent	0.672		
60. Not familiar with the policies of sickness relief	0.648		
57. The older adult interfering too much with the family life	0.598		
Factor 2: Enduring treatment ordeals		12.351%	0.918
14. Worry that the child is too young to bear the surgery	0.869		
15. Worry about the effect of surgery	0.814		
16. Worry about surgical anesthesia side-effect on the child’s health	0.796		
21. Worry that the child is afraid of the unfamiliar environment in the hospital	0.731		
10. Seeing the surgical incision on the child	0.723		
11. Seeing the child with a tube or monitor attached after surgery	0.719		
17. Worry that the child cannot obtain considerate caring in the intensive care unit	0.676		
7. Too long duration of the Child’s surgery	0.643		
Factor 3: Dilemma in the family coping process		13.014%	0.940
31. Declining family income	0.841		
30. Family living expenses larger during their child’s hospitalization	0.833		
Factor 3. High cost of daily medical care	0.817		
46. Caring for the child interferes with work	0.813		
50. Families have to make sacrifices for the children	0.790		
47. Need to balance work and family	0.761		
45. Caring for the child interferes with personal life	0.743		
44. Family being in debt before the child fell ill	0.713		
33. Caring for other family members	0.685		
29. High cost of surgery	0.669		
Factor 4: Confusion in the understanding of the disease		6.583%	0.774
1. Cannot understand why my child is sick	0.726		
3. Do not know the severity of the child’s illness	0.708		
4. Do not know how the child’s illness affects the family	0.647		
2. Idea that consider it may be my own fault makes the child sick	0.544		
Factor 5: Family resilience		9.378%	0.867
55. Siblings being more independent	0.826		
53. Family members being more considerate of each other	0.783		
56. Siblings taking care of sick children	0.762		
54. Parents working harder to make money	0.616		
Factor 6: Unusual growth track of the child owing to the disease		11.983%	0.907
21. Child’s movement restriction	−0.800		
23. Child’s activity limitation because of the worries about his/her health	−0.774		
25. Child often having a cold	−0.733		
37. Worry that the child will feel inferior	−0.684		
24. Worry that the child’s crying could lead to a flare-up	−0.683		
26. Slow physical development of the child	−0.676		
19. Do not know how to explain disease to the child	−0.651		
40. Worry about marriage and fertility problem of the child	−0.640		

### Confirmatory factor analysis

The CFA with the maximum-likelihood estimation method was used to verify the model. The fitness indices of the model were as follows: CMIN/DF = 2.067, RMSEA = 0.057, RMR = 0.095, CFI = 0.921, IFI = 0.921, TLI = 0.913, which indicated a good fit between the model and the data (38, 39, 41). All indices provided confirmatory evidence for the factor structure.

### Criterion-related validity

The correlation coefficient between CHD Children’s Family Stressor Scale and SAS was *r* = 0.504 (*p* < 0.001), which indicated that the criterion-related validity of CHD Children’s Family Stressor Scale was good.

### Internal consistency reliability

Cronbach’s α coefficient value of the scale level is 0.945, and Cronbach’s α coefficient values of the six dimensions in the scale are 0.906, 0.918, 0.940, 0.907, 0.774, and 0.867, respectively, which indicate a satisfactory internal consistency reliability of the scale as a whole and each dimension (40).

### Measurement invariance

The model fit indices of all baseline models, for fathers and mothers separately, are summarized in Table 4. The invariance model is considered acceptable when the value of the CFI difference (ΔCFI) is below 0.010 (42). The tests of metric invariance (ΔCFI = 0), strong invariance (ΔCFI = −0.001) and scalar invariance (ΔCFI = −0.002) showed good fit, which indicated that the scale factor structure reached measurement invariance across the sample of fathers and mothers.

**Table 4:** Measurement invariance of the family stressor scale for children with CHD.

Model	df	χ^2^	*p*	RMSEA	GFI	CFI	TLI	RMR	Δχ^2^	ΔRMSEA	ΔCFI
Configural invariance	1,504	3390.25	<0.001	0.044	0.792	0.910	0.902	0.113			
Metric invariance	1,539	3426.634	<0.001	0.044	0.791	0.910	0.904	0.120	36.384	−0.001	0
Strong invariance	1,560	3459.158	<0.001	0.043	0.788	0.909	0.904	0.129	68.908	−0.001	−0.001
Scalar invariance	1,613	3535.963	<0.001	0.043	0.785	0.908	0.906	0.129	145.713	−0.001	−0.002

## Discussion

To the knowledge of the authors, this is the first study both to develop and undertake a detailed validation of a scale to assess the stressors of families with children diagnosed with CHD. It consists of 41 items, which are organized into six subscales (ambiguity, both intra-family and social, enduring treatment ordeals, dilemma in the family coping process, confusion in the understanding of the disease, and family resilience and unusual growth track of the child owing to the disease). When constructing the item pool, qualitative research was conducted to clarify the structural composition of the scale and operationally define the concepts measured guided by the double ABCX model of family stress and adaptation. Previous related studies on parental stressors of children with CHD (10, 43, 44) and related stressor scales (20, 22, 24–26, 45, 46) were combined to develop the items. These items were generated from the perspective of family members of children with CHD and verified by the published literature in the field of expertise. This approach not only conforms to the principles of measurement tool development (16, 47) but also ensures the content validity of the tool. The items were verified using the expert consensus meeting method and the expert consultation method. Experts from different majors in the field of CHD health management provided suggestions for item optimization from different perspectives, which strengthens the face validity and content validity of the scale.

In assessing the psychometric properties of the CHD children’s family stressor scale, exploratory and confirmatory factor analyses supported the evidence for the construct validity of the scale. This scale extracted six factors through EFA, and the cumulative explanatory variables of the six factors were 61.085%. The obtained model was basically consistent with the double ABCX model of family stress and adaptation. The model was tested using CFA, which showed a good fit between the model and the data. An RMSEA less than 0.06 is considered a close fit (41), the CFI, IFI, and TLI of the obtained model are all greater than 0.90, and the values of CMIN/DF, RMSEA, and RMR also supported the acceptable fit of the model (48). The correlation coefficient between CHD children’s family stressor scale and SAS was *r* = 0.504 (*p* < 0.001), which indicated that the criterion-related validity of CHD children’s family stressor scale was good. Cronbach’s α coefficient of each dimension in this scale is greater than 0.7, and Cronbach’s α coefficient value of the scale level is greater than 0.9, which proves that the scale has relatively ideal internal consistency reliability. By sub-analyses, we found that there was no statistical difference in the mean scores of the CHD Children’s Family Stressor Scale among different sample groups, which indicated that the scale can be used across populations (i.e., fathers and mothers).

From the above results, it can be seen that the CHD children’s family stressor scale is an effective and reliable tool for evaluating the stressors of CHD children’s families. Numerous studies have shown that families of children with CHD suffer from physical, psychological and economic torment during the long process from diagnosis to surgery, and they face stressors from different aspects (49). How to better manage stress is one of the important issues faced by families of children with CHD. In the practice of stress management in families with CHD children, the scale can be utilized as an effective assessment tool for healthcare professionals to understand the stressors of families with CHD children and provide targeted interventions to relieve family stress.

### Limitations

This study has several limitations. First, participants were mainly recruited primarily from hospitals in the southwest of China, so these data may not be generalizable nationwide. Second, the recruited participants were mainly parents of children with CHD, lacking the perspectives of other family members, which may lead to limitations in the study content. Finally, the scale has only large-scale investigation once, and its convergent/divergent validity and test–retest reliability were not examined, and the effect practical application of the scale can be further verified in future studies. Therefore, in future studies, it is necessary to expand the sample collection area and include other family members of children with CHD, such as siblings and grandparents.

## Conclusion

We finally obtained the CHD Children’s Family Stressor Scale, with six dimensions and 41 items. The six dimensions are ambiguity, both intra-family and social, enduring treatment orders, dilemma in the family coping process, confusion in the understanding of the disease, family resilience and unusual growth track of the child owing to the disease. The scale has been tested to be a reliable and valid tool for assessing the stressors of families with CHD children. Although the above limitations, preliminary findings suggest that this newly developed tool has good reliability and validity. In addition, criterion validity based on correlation with other validated tools is also appropriate. Therefore, it is recommended that this tool be used in clinical practice to assess stressors in families of children with CHD and provide targeted interventions for stress management in families with children with CHD.

## Data availability statement

The original contributions presented in the study are included in the article/supplementary material, further inquiries can be directed to the corresponding author.

## Author contributions

YZ: Data curation, Formal analysis, Methodology, Software, Writing – original draft. HZ: Formal analysis, Methodology, Writing – review & editing. YB: Data curation, Investigation, Writing – review & editing. ZC: Data curation, Writing – review & editing. YW: Investigation, Writing – review & editing. QH: Formal analysis, Methodology, Writing – review & editing. MY: Supervision, Writing – review & editing. WW: Writing – review & editing. LD: Supervision, Writing – review & editing. FM: Funding acquisition, Investigation, Methodology, Supervision, Writing – review & editing.
