# Corrigendum: Development and validation of a questionnaire to measure the congenital heart disease of children's family stressor

**DOI:** 10.3389/fpubh.2024.1541193

**Published:** 2024-12-18

**Authors:** Yi Zhang, Hang Zhou, Yangjuan Bai, Zhisong Chen, Yanjiao Wang, Qiulan Hu, Mingfang Yang, Wei We, Lan Ding, Fang Ma

**Affiliations:** ^1^Department of Nursing, Kunming Municipal Hospital of Traditional Chinese Medicine, Kunming, China; ^2^Department of Clinical Psychology, Yunnan Provincial Hospital of Infectious Disease, Kunming, China; ^3^Cardiology Department, The First Affiliated Hospital of Kunming Medical University, Kunming, China; ^4^Psychiatric Department, The First Affiliated Hospital of Kunming Medical University, Kunming, China; ^5^ICU in Geriatric Department, The First Affiliated Hospital of Kunming Medical University, Kunming, China; ^6^Urology Department, The First Affiliated Hospital of Kunming Medical University, Kunming, China; ^7^Neurosurgery Department, The First Affiliated Hospital of Kunming Medical University, Kunming, China; ^8^General Surgery Department, The First Affiliated Hospital of Kunming Medical University, Kunming, China; ^9^Department of Nursing, The First Affiliated Hospital of Kunming Medical University, Kunming, China

**Keywords:** congenital heart disease, validity, reliability, family, stressor

In the published article, there was an error in the affiliation for author **Fang Ma**. The affiliation of this author should be changed from “Department of Nursing, Kunming Municipal Hospital of Traditional Chinese Medicine, Kunming, China” to “Department of Nursing, The First Affiliated Hospital of Kunming Medical University, Kunming, China”.

The authors apologize for this error and state that this does not change the scientific conclusions of the article in any way. The original article has been updated.

